# Silent cerebral infarct in sickle cell anemia patients of southern Turkey

**DOI:** 10.3906/sag-2003-192

**Published:** 2020-12-17

**Authors:** Ezgi NAFİLE SAYMAN, Göksel LEBLEBİSATAN, Şerife LEBLEBİSATAN, Kenan BIÇAKÇI, Yurdanur KILINÇ, Adnan BARUTÇU

**Affiliations:** 1 Department of Pediatrics, Faculty of Medicine, Çukurova University, Adana Turkey; 2 Department of PediatricHematology, Faculty of Medicine, Çukurova University, Adana Turkey; 3 Department of Radiology, Adana City Hospital, Adana Turkey; 4 Department of Radiology, Faculty of Medicine, Çukurova University, Adana Turkey

**Keywords:** Sickle cell anemia, silent cerebral infarct, stroke, magnetic resonance imaging, children

## Abstract

**Background/aim:**

Silent cerebral infarct (SCI) is an ischemic lesion seen before clinical signs of brain infarct and ischemic changes in brain tissue. This study aimed to detect SCI with noninvasive methods and to determine related risk factors in patients with sickle cell anemia (SCA).

**Materials and methods:**

Fifty-four SCA patients who had no history of cerebral infarct and whose neurological examinations were normal were included in this study. Brain magnetic resonance imaging (MRI) and diffusion MRI were taken and the acquired data was compared statistically.

**Results:**

SCI was detected in 11.1% (6/54) of the patients. No statistical differences in age, sex, physical examination findings, or treatments were detected between the 2 groups (with and without SCI). When examined in terms of HbS, the median (min–max) value in SCI-positive patients was 85.4 (80.5–92.1); the median value was 77.2 (49.0–96.7) in SCI-negative patients. The HbS values of the SCI group were statistically significantly higher than those of the group without SCI (P = 0.014). Patients with the HbSS or HbSβ0 genotypes had a significantly higher prevalence of SCI when compared with other sickle cell syndromes (P = 0.038).

**Conclusion:**

SCI is not uncommon among SCA patients in Turkey. The presence of homozygote HbSS/Sβ0 genotype, high MCV, and HbS are risk factors for SCI.

## 1. Introduction

Sickle cell anemia (SCA) is widely distributed in Africa and the Mediterranean region [1,2]. Sickle hemoglobin (Hb) is the most common abnormal Hb in Turkey, with a frequency of 0.37%–0.60%, and an HbS heterozygosity of 3%–44% in the Çukurova region [3].

Central nervous system (CNS) complications, such as stroke, are among the most devastating manifestations of SCA. Silent cerebral infarcts (SCI) are detected by magnetic resonance imaging procedures but do not cause abnormalities on neurological examination. These ischemic lesions seem to be related to the recurrence of an infarct; either clinical stroke or SCI are associated with transient ischemic attack (TIA), and also related with mild mental retardation and future hemorrhagic stroke [4,5].

A plethora of literature is available on SCI in children, but much less research is available where SCA is concerned, and rarely includes studies from Turkey. Our study, conducted in southern Turkey, aimed to detect SCI by noninvasive imaging methods in patients with SCA before any possible serious complications, such as CNS events, as well as to identify the predisposing factors and shed light on treatment protocols.

## 2. Materials and methods

Fifty-four SCA patients,including children, adolescents, and young adults(26 female, 28 male), who had no previous stroke history or abnormal neurological findings and were followed-up with by the PediatricHematology Clinic of Çukurova University, were included in the study. The patients were aged between 5 and 31 years. These patients underwent brain magnetic resonance imaging (MRI) and diffusion MRI for SCI.

A detailed evaluation of the patients’ history (treatments, crises, erythrocyte transfusions, or apheresis) was noted. Exclusion criteria included abnormal findings on neurological examination, a painful or hemolytic crisis during outpatient clinic control, a history of painful or hemolytic crisis within the past 3 months, a history of infection within the last month, or a blood transfusion within the last 3 months. Patients without any neurological findings on physical examination were included in the study.

Verbal and written informed consent about the purpose of the examinations was obtained from patients aged >18 years, or from patients’ families (for patients <18 years).

Conventional MRI and diffusion MRI of each patient’s brain was performed at our hospital, using a 1.5 Tesla MRI device located in the Department of Radiodiagnostics. During MRI imaging, the patient was not given any anesthetic agent. The MRI data were reported by members of the Radiodiagnostics Academic Staff.

Cerebral silent infarcts were identified as hyperintense focal lesions in T2 and hypointense focal lesions in T1 that were less than 15mm and greater than 3mm on MRI, which enables the detection of lesions in regions such as the basal ganglia that are indistinguishable in computed tomography (CT) [6–8].

Ethics committee approval (Session No.40, Decision No. 16, date: 06.03.2015) was obtained from the Non-Interventional Clinical Research Ethics Committee of Çukurova University Faculty of Medicine.

Statistical analysis

The statistical package for the social sciences, which is known as SPSS (IBM Corp., Armonk, NY, USA) v. 23.0 program was used for statistical analysis of the data. Categorical measurements were summarized as numbers and percentages, while continuous measurements were summarized as median and minimum–maximum. Fischer’s exact test was used to compare categorical variables. The Shapiro–Wilk test was used to determine whether the parameters in the study showed normal distribution. In comparing continuous measurements between groups, distributions were checked, and the Mann–Whitney U test was used for parameters that did not show normal distribution. In the study, based on the HbS value according to the MRI results, the sensitivity and specificity values ​​were calculated according to the SCI positivity and the area under the ROC curve was examined. Statistical significance level was established as 0.05 in all tests.

## 3. Results

Of the 54 SCA patients undergoing follow-up and treatment at the Pediatric Hematology Department, 6 patients had SCI based on the brain MRI and diffusion MRI examinations. When the patients are distributed by age group, 3 of the patients were under the age of 8, 34 were between the ages of 8 and 16, and 17 were older than 16 years. Five (14.7%) of the patients in the 8–16 group and 1 (5.9%) of the patients over the age of 16 were SCI (+).When examining the presence of SCI within these age groups, there was no statistically significant difference among the age groups (P = 0.756). Distribution of patients by age group is shown in Table 1.

**Table 1:** Distribution of patients by age groups.

Age groups	SCI (+) (n = 6)	SCI (–) (n = 48)	Total (n = 54)	P*
	n (%)	n (%)	n (%)	0.756
< 8 years	0 (0)	3 (100)	3 (100)
8–16 years	5 (14.7)	29 (85.3)	34 (100)
>16 years	1 (5.9)	16 (94.1)	17 (100)

*Fisher’s exact test

**Patient 1 (S.D.):** A 15-year-old female patient was regularly using folic acid and 18 mg/kg of hydroxyurea. Her liver was normal in size, but she had developed otosplenectomy, regularly received blood transfusions, and had a history of more than 5 painful crises per year. Without a blood transfusion, her HbS level was 84.7%.

**Figure 1:**
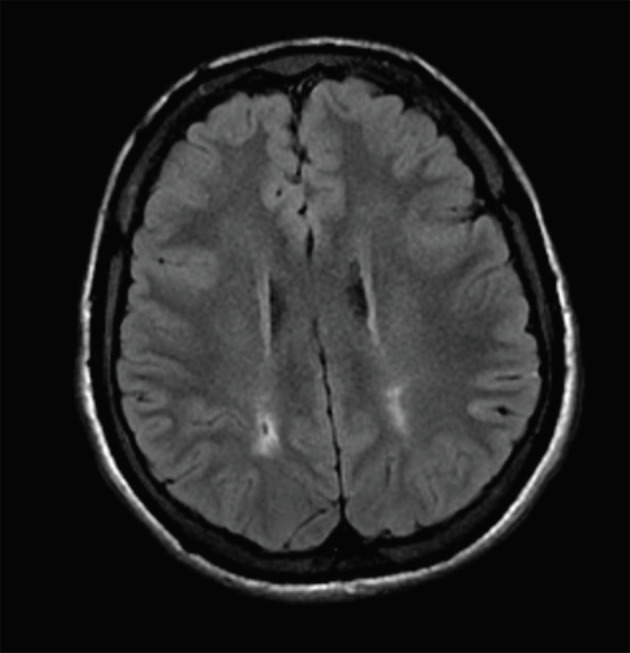
Axial FLAIR MR image. Biparietal white matter hyperintensities with cystic changes at right.

**Figure 2:**
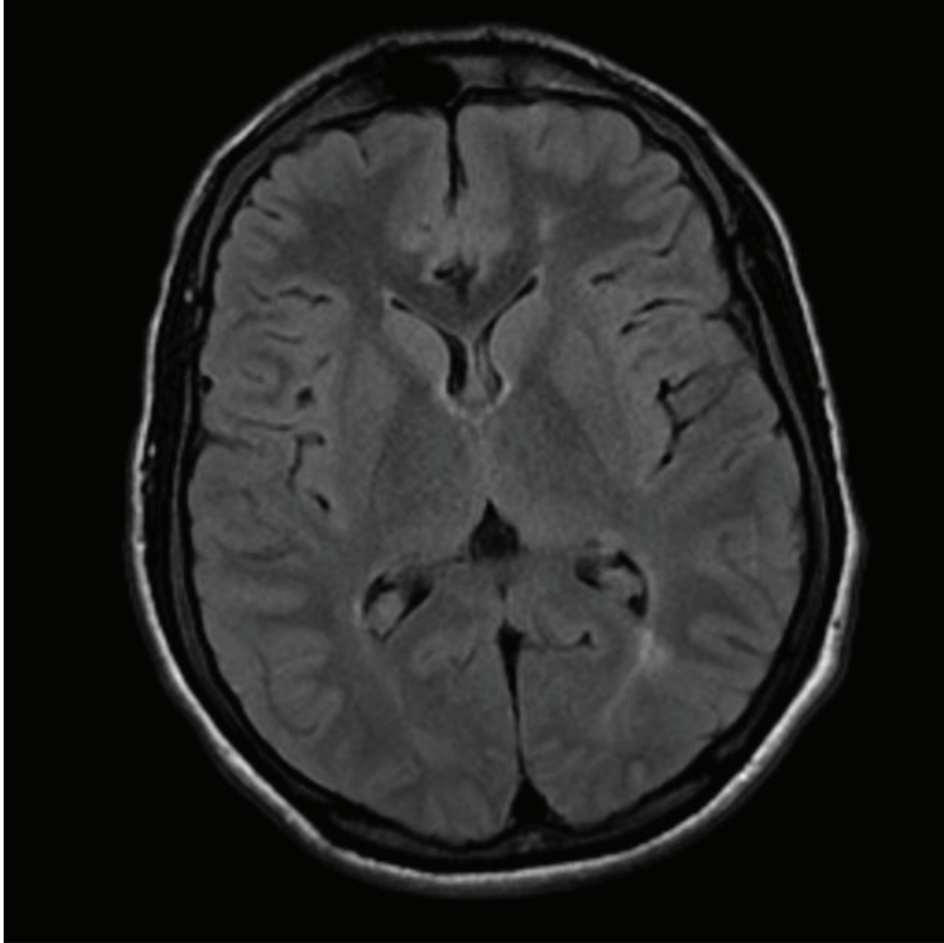
Silent cerebral infarctions in the left frontal and parietal white matter.

**Patient 2 (S.G.)** : A 19-year-old male patient was regularly using folic acid, zinc sulfate, deferasirox, and 15 mg/kg hydroxyurea. He had a normal-sized liver prior surgical splenectomy, a history of painful crises, and acute chest syndrome. His HbS level was 90.5% (Figures 1 and 2).

**Patient 3 (F.D)** : A 16-year-old male patient was regularly using folic acid, zinc sulfate, and 8 mg/kg of hydroxyurea. Hepatomegaly was present. The spleen was normal in size, and the patient had a history of more than 5 painful crises per year. His HbS level was 83.3%.

**Patient 4 (S.B)** : A 16-year-old female patient was regularly using folic acid. She had a normal-sized liver, prior surgical splenectomy, and had only had 1 painful crisis event. Her HbS level was 80.5%.

**Patient 5 (Ç.S.T.)** : An 8-year-old male patient was regularly using folic acid, zinc sulfate, and 22 mg/kg of hydroxyurea. Hepatomegaly was present, the spleen was normal in size, and there was a history of acute chest pain and painful crises. His HbS level was 92.1%.

**Patient 6 (D.T.)** : A 16-year-old male patient regularly used folic acid and zinc sulfate. The liver was normal in size, and autosplenectomy had developed. He had a history of painful crisis and priapism. His HbS level was 86.1%.

The hemoglobin electrophoresis results of the patients were examined. In terms of HbS, the median (min–max) value of SCI-positive patients was 85.4 (80.5–92.1), and 77.2 (49–96.7) in SCI-negative patients. The HbS values of the SCI group was statistically significantly higher than that of the group without SCI (P= 0.014). The median (min–max) HbF values of the groups with and without SCI were 11.1 (3.5–17.7) and 15.8 (4.7–40.9). When these 2 groups were compared in terms of HbF, no statistically significant difference was found (P = 0.073) (Table 2).

**Table 2:** Hemoglobin electrophoresis results.

	SCI (+)(n = 6)	SCI (–)(n = 48)	P*
	Median (min–max)	Median (min–max)	
HbA	-	15.6 (1.6–42.6)	-
HbS	85.4 (80.5–92.1)	77.2 (49.0–96.7)	0.014
HbF	11.1 (3.5–17.7)	15.8 (4.7–40.9)	0.073
MCV	94.6 (85.9–115.0)	87.0 (57.7–115.5)	0.035

*Mann–Whitney U Test

The median (min–max) MCV values were 94.6 (85.9–115) and 87.0 (57.7–115.5) fL for the patients with and without SCI, respectively. When the MCV values of the hemogram were examined, the MCV values of the patients with SCI were significantly higher than those of the patients without SCI (P = 0.035) (Table 2).

When the patients were grouped according to Hb electrophoresis results and Hb mutations, 33 patients had HbSS or HbSβ0 mutations, and 21 patients had Sβ+ or other sicklecell syndromes. SCI was present in 6 patients in the HbSS/Sβ0group, and none of the patients was in the Sβ+ or other sickle cell syndrome group (P = 0.038) (Table 3).

**Table 3:** Distribution of patients according to the results of hemoglobin mutations.

Hemoglobin mutations	SCI (+)(n = 6)	SCI (–)(n = 48)	Total(n = 54)	P*
	n (%)	n (%)	n (%)	0.038
HbSS and HbSβ0	6 (18.2)	27 (81.8)	33 (100)
Sβ+ or other mutations	0 (0)	21 (100)	21 (100)

*Fisher’s exact test

When the drugs used by the patients with sickle cell anemia were investigated, approximately 80% of the patients were using hydroxyurea (43 people). However, in terms of the presence of SCI, no statistical difference was found between them (P =0.590). Comparison of the groups in terms of drugs used by patients is summarized in Table 4.

**Table 4:** Comparison of groups in terms of drugs used by patients.

	Treatment given	SCI (+)(n = 6)	SCI (–)(n = 48)	Total(n = 54)	P*
		n (%)	n (%)	n (%)	
Hydroxyurea	Yes	4 (9.3)	39 (90.7)	43 (100)	0.590
	No	2 (18.2)	9 (81.8)	11 (100)	
Deferasirox	Yes	1 (33.3)	2 (66.7)	3 (100)	0.303
	No	5 (9.8)	46 (90.2)	51 (100)	
Folic acid	Yes	6 (12)	44 (88)	50 (100)	0.672
	No	0 (0)	4 (100)	4 (100)	
Zinc	Yes	4 (10.3)	35 (89.7)	39 (100)	0.747
	No	2 (13.3)	13 (86.7)	15 (100)	

*Fisher’s exact test

**Figure 3:**
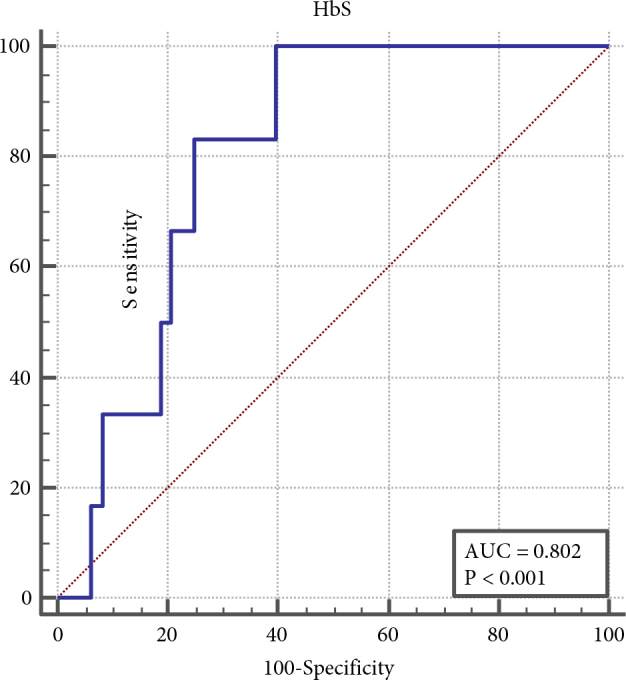
ROC curve to determine SCI presence according to the HbS measurement.

ROC analysis was performed in terms of the presence of a cut-off value in determining the statistically significant HbS measurement in the presence of SCI. As a result of ROC analysis for HbS, the area under ROC is 0.802; cut-off was detected as 79.6 (P= 0.0001). In other words, the probability of having SCI was 80% according to the cut-off value determined. When the patient’s HbS measurement value was determined to be above 79.6 as a result of the MRI, SCI was expected with 80.2% probability, 100% sensitivity, and 60.4% specificity (Table 5, Figure 3).

**Table 5:** ROC curve analysis results to determine the presence of SCI according to HbS measurement.

	HbS
AUC	0.802
95%–Cl%	0.671–0.898
Cut-off	>79.6
Sensitivity (%)	100,0
95%– Cl%	54.1–100.0
Specificity (%)	60.42
95%–Cl%	45.3–74.2
PPV	24.0
NPV	100.0
+LR	2.53
–LR	0.0
P*	0.0001

*ROC curve test

## 4. Discussion

One of the most serious complications of SCA is CNS events. SCI is defined as infarction detected by imaging methods in cases without neurological symptoms. In patients with SCA, SCI is seen approximately twice as often as clinical stroke in the same population [9]. Silent infarcts are mostly associated with occlusion of small vessels and are localized in deep white matter or basal ganglia [10]. Silent infarcts may be a sign of increased risk of clinical stroke or new and widespread silent infarcts. Neurological complications may progress to mild mental retardation due to silent infarcts, transient ischemic attacks, severe ischemic attack, or hemorrhagic infarction [11,12]. Aplastic crisis, acute splenic sequestration, and pulmonary sequestration are risk factors for these infarcts. Neurocognitive deficits have been shown in patients with silent infarction. Treatment approaches to prevent silent infarcts are uncertain [13].

In a study by Pegelow et al., the rate of SCI was 22% in SCA patients [14]. In 2010, Bernaudin et al. reported that SCI was the most common cerebrovascular disease seen in SCA patients: 28% in childhood before the age of 6 years [15]. Kwiatkowski et al. found a prevalence of 37% in SCA patients before the age of 14 years [16].

A recently published article found that 30% of SCA patients had SCI before the age of 18 years. In the same study, the annual incidence in childhood was found to be around 1%, especially in patients with SCA, with regular follow-up and necessary prophylaxis [17].

SCI can start in infancy. Its frequency increases with age and can be seen in nearly half of patients during adolescence. In a study conducted in patients with adult SCA, the incidence of SCI was reported to be up to 60% [18]. Based on the current literature, it can be concluded that SCI is seen in SCA patients in childhood and can reach serious proportions at later ages. In our study of patients aged 5 to 31 years, the SCI rate was 11.1%.

This SCI rate is the lowest level to be mentioned in the literature to date, and is not as high as in the patients who did not receive the necessary prophylaxis treatment for the possible follow-up problems. Our study group consisted of 3 children, 29 adolescents, and 17 young adults. Since most of the patients were adolescents and the evaluation of the patients was based on immediate follow-up results without a specific follow-up period, we may not have been able to identify a particular association between the age group and SCI.

In an SCI transfusion trial with 814 patients aged 5 to 15 years without stroke or seizure history, brain MRI scans were performed to investigate the correlation of SCI presence with some clinical and laboratory variables. In that study, male sex, low Hb level, and high systolic blood pressure were found to be statistically significant risk factors for SCI [19]. In our study, 6 patients with SCI were male, but 4 of these patients were not statistically different in terms of sex.

Other studies have identified some of the risk factors for SCI as an increased white blood cell count, presence of seizures, low Hb values, presence of cerebral artery stenosis in cerebral MRI angiographs, a low number of painful crises, and the Senegal haplotype [20]. In a recent review by the American Hematology Society, SCI was found in patients with SCA; the HbSS or HbSβ0 mutation-carrying group was found to be twice as likely to have SCI as other sickle cell syndromes, such as HbSC and HbSβ+[17]. Similar to the results in the literature, in our study, patients with HbSS or HbSβ0 mutation had a significantly higher prevalence of SCI than HbSβ+ or other sickle-cell syndromes.

SCI is not associated with serious neurological complaints but is linked with cognitive deficits. In a study conducted by Kawadler et al. in 2016, IQ tests were performed in 3 different groups. The IQ of patients with stroke-experienced SCA was 10 points lower than that of the SCI population, 6 points lower in the SCA group with SCI than in the SCA group without SCI, and 7 points lower in the SCA group than in the normal population [21].

In 2014, Najibah et al. emphasized the importance of hydroxyurea therapy for primary stroke prevention in patients with SCA. In that work, a group of patients with predicted stroke and silent infarction risk through increased flow velocity in transcranial Doppler ultrasonography was treated with hydroxyurea for 3 months [22]. At the end of this period, it was determined that the flow velocities of the TCD measurements of the patients were significantly decreased compared with the pretreatment data. There was a significant increase in the MCV and HbF values in blood tests before and after treatment. Additionally, as part of the treatment strategy, a regular blood transfusion for at least 1 year was recommended in the patients with abnormal TCD measurements [22]. In our patient group, 4 of 6 patients with SCI were receiving regular hydroxyurea treatment, while 2 of these patients were diagnosed with SCI and hydroxyurea treatment then followed. Although MCV elevation has been reported to increase due to hydroxyurea use, in our study, there was a statistically significant increase in the MCV values in patients with SCI compared to patients in the group without SCI. When SCI- and MCV-related publications were considered, the use of hydroxyurea for prophylaxis of SCI was found to be associated with significantly higher MCV in patients than in patients without SCI. Our results were incompatible with this and other literature data.

In our study, the clinical and laboratory investigations of the patients revealed the presence of homozygous HbSS/Sβ0 genotype, MCV elevation, and HbS elevation as risk factors. Although some of the results were consistent with the literature, others were not. However, our study had several limitations, including the number of patients, the broad age distribution of the patients, and the inclusion of patients with instantaneous values based on the eligibility criteria, not only during a regular follow-up period.

Our study is one of the few to examine the presence of SCI in children with SCA in Turkey and different ethnicities. Most of the previous studies involve patients of African origin. The presence of SCI is thought to be a predictor of an early stroke in SCA patients. More extensive studies are needed in multiple centres in Turkey in order to establish an exact relationship between the clinical and laboratory data of children with SCA and the presence of SCI.

## Informed Consent

The authors assert that all procedures contributing to this work comply with the ethical standards of the relevant national guidelines on human experimentation and with the Helsinki Declaration. Ethics committee approval (Session No. 40, Decision No. 16, date: 06.03.2015) was obtained from the Non-Interventional Clinical Research Ethics Committee of Cukurova University Faculty of Medicine.

The patients and their families were informed about the examinations and about the study. In addition, verbal and written informed consent was obtained from all patients and their families.
